# Validity and Utility of Early Parameters in TEG6s Platelet Mapping to Assess the Coagulation Status During Cardiovascular Surgery With Cardiopulmonary Bypass

**DOI:** 10.7759/cureus.38044

**Published:** 2023-04-24

**Authors:** Yusuke Yoshikawa, Makishi Maeda, Sho Ohno, Kanako Takahashi, Yasuaki Sawashita, Tomoki Hirahata, Yutaka Iba, Nobuyoshi Kawaharada, Mitsutaka Edanaga, Michiaki Yamakage

**Affiliations:** 1 Department of Anaesthesiology, Sapporo Medical University, Sapporo, JPN; 2 Department of Cardiovascular Surgery, Sapporo Medical University, Sapporo, JPN

**Keywords:** coagulation, cardiac surgery, cardiopulmonary bypass, platelet mapping, teg6s

## Abstract

Background

The aim of this retrospective observational study was to explore the early predictive parameters for maximum amplitudein the kaolin with heparinase (HKH) assay (MA_HKH_) of TEG6s Platelet Mapping in cardiovascular surgery including cardiopulmonary bypass (CPB) period. The relationship between each parameter of the assay and laboratory data was also assessed.

Methods

We included the patients who underwent TEG6s Platelet Mapping during cardiovascular surgery under CPB between November 2021 and May 2022. The correlation between MA_HKH_ and the early parameters was assessed. The association between each parameter of Platelet Mapping and a combination of fibrinogen concentration > 150 mg/dL and platelet count > 100,000µL was also evaluated by the receiver operating characteristic (ROC) curve.

Results

In 23 patients who underwent TEG6s Platelet Mapping during the study period, 62 HKH assay data including 59 pairs of data (HKH assay and laboratory data) were analyzed. K and angle, but not R, were significantly correlated with MA_HKH _(r [95% CI]: -0.90 [-0.94, -0.83], p < 0.0001 for K, and 0.87 [0.79, 0.92], p < 0.0001 for angle). Furthermore, ROC curves suggested that these parameters predicted a combination of fibrinogen concentration > 150 mg/dL and platelet count > 100,000/µL with high accuracy. Similar results were confirmed in the heparinized blood samples obtained during CPB.

Conclusion

These findings suggest that not only MA_KHK _but also K and angle, which are early parameters in the HKH assay, provide clinically significant information that will facilitate rapid decision-making regarding coagulation strategies during cardiovascular surgery including the CPB period.

## Introduction

Cardiovascular surgery with cardiopulmonary bypass (CPB) induces significant consumption coagulopathy in a multifactorial manner [1,2]. Current clinical guidelines [3-7] strongly suggest the use of point-of-care (POC) monitoring for guiding transfusion strategies of blood and blood products during cardiovascular surgery. Coagulation is a complex process involving various types of cells, platelets, and coagulation factors [8]. Therefore, it is important to assess the overall coagulation status of whole blood using POC monitoring. Furthermore, it is preferable to assess blood coagulation status using rapid monitoring measured within a short period of time throughout surgery, including the CPB period, to guide rapid practical decision-making in blood coagulation management [4].

TEG6s Platelet Mapping (Haemonetics Co., Braintree, MA) is a POC monitoring method based on a viscoelastic coagulation assay and was originally developed for the analysis of adenosine diphosphate (ADP)- and arachidonic acid (AA)-induced platelet function, especially in patients receiving antiplatelet medication. However, this monitoring also assesses the overall coagulation ability triggered by kaolin utilizing the heparin reversal (heparinase (HKH) assay), as well as independent fibrin formation (ActF assay). These assays allow the evaluation of the blood coagulation status even in heparinized blood samples during CPB. According to a recent report [9], the ActF assay can predict serum fibrinogen concentration even in heparinized blood samples. However, evidence for the utility of the HKH assay is very limited in cardiac surgery, especially during CPB.

In addition to maximum amplitude (MA), which has been reported to be associated with postoperative bleeding [10], the HKH assay also measures the R, K, and angle, which are measured in the earlier phases of thromboelastography. These early parameters are expected to correlate with MA (MA_HKH_) measured in the late phase of thromboelastography; however, these correlations have not been validated. This relationship is critical to guiding timely decision-making for coagulation management during cardiovascular surgery.

We hypothesized that the early parameters were strongly associated with MA_HKH_ in TEG6s Platelet Mapping. Clarifying the relationship between R, K, angle, and MA_HKH_ in the HKH assay was the goal of the current study in order to identify early predictive parameters for MA_HKH_ including in the CPB period. Furthermore, because the HKH assay represents the overall coagulation state, the ability of each parameter to predict the combination of fibrinogen > 150 mg/dL [11,12] and platelet count > 100,000/µL [12,13] was also examined.

## Materials and methods

The data supporting the findings of this study are available from the corresponding author upon reasonable request.

Patients and ethics approval

Adult patients (≥ 18 years old) who underwent cardiovascular surgery with CPB between November 2021 and May 2022 were enrolled in this study. Patients who did not undergo TEG6s Platelet Mapping assay during surgery were excluded. This retrospective study was approved by the local institutional review board (approval number: 343-63) and was conducted in accordance with the Declaration of Helsinki. The requirement for written informed consent was waived because of the retrospective nature of the study and because the analysis was conducted anonymously. This trial was registered in the Japan Registry of Clinical Trials (jRCT1012220015).

Clinical management

All antiplatelet drugs were discontinued preoperatively according to local institutional standards and replaced with unfractionated heparin in elective surgeries. All patients received general anesthesia and cardiac surgery with CPB according to local institutional standards. Briefly, an arterial catheter (20 gauge) was inserted into the right radial artery before the induction of general anesthesia. After induction of general anesthesia, the trachea was intubated, and a central venous catheter was inserted via the internal right jugular vein. A pulmonary artery catheter was also placed in some patients, depending on the anesthesiologist’s decision. Complete anticoagulation was achieved with 300 IU/kg of unfractionated heparin for CPB. The activated clotting time (ACT) was monitored to maintain ACT > 480 s during CPB, and additional heparin was administered as needed. All patients received 100 mg betamethasone and 1000 mg tranexamic acid prior to CPB initiation. The standard CPB circuit consists of a venous reservoir, cardiotomy reservoir, centrifugal pump, membrane oxygenator, and arterial filter. Deep hypothermic circulatory arrest and selective cerebral perfusion were performed, as needed. The priming volume was 800-1500 mL, and the priming solution consisted of Ringer’s solution, d-mannitol, and 6% hydroxyethyl starch 130/0.4.

Sample collection

Blood sampling was performed during the preoperative period (within seven days before surgery or just before induction of general anesthesia), during CPB (typically during aortic unclamping), and at the end of surgery as a part of routine practice in our institute. Serum fibrinogen concentration and platelet count were measured in the clinical laboratory using blood samples collected in VACUETTE® blood collection tubes with 3.2% sodium citrate (3 mL) and VACUETTE® blood collection tubes with EDTA (2 mL) (Greiner Bio-One, Monroe, NC, USA), respectively. POC monitoring was also performed to assess the coagulation state in whole blood using the HKH assay of TEG6s Platelet Mapping using blood samples collected in heparinized BD Microtainer® blood collection tubes (400 µL) or BD Vacutainer® blood collection tubes (4 mL) (Becton Dickinson, Franklin Lakes, NJ).

TEG6s Platelet Mapping

**Figure 1:**
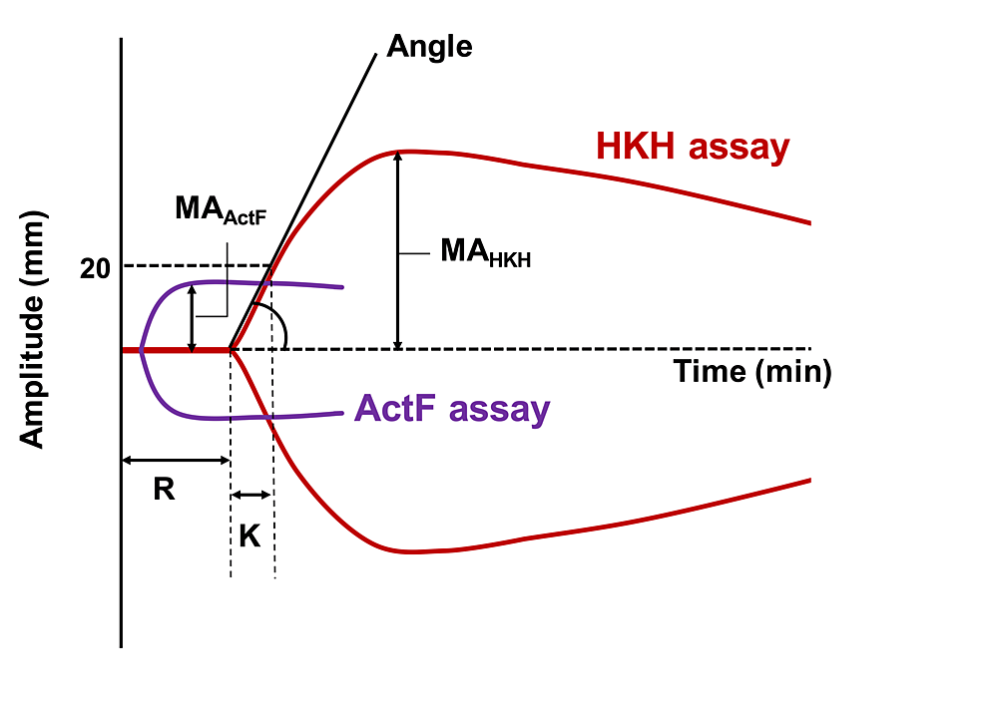
Scheme of HKH and ActF assays in TEG6s Platelet Mapping

Blood samples were applied to the TEG6s Platelet Mapping cartridge, and data from the HKH and ActF assays were collected (Figure 1).

In the HKH assay, the effect of heparin is antagonized by HKH, and thromboelastography triggered by kaolin distinguishes the R (minute), K (second), angle (degree), and MA (mm). R, K, and angle are the rapid parameters (measured typically within 10 minutes) in the HKH assay of TEG6s Platelet Mapping, where R is the time required for the amplitude to reach 2 mm (initial fibrin formation), K is the time from the start of clot formation to a point with 20 mm of amplitude (amplification), and the angle is the angle of the tangent line at the middle of the R and K points (thrombin burst) [14,15]. Together, these parameters represent the speed of clot formation. MA is measured in the late phase of thromboelastography (usually 20-40 minutes according to the coagulation state), which represents the strength of the clot. In the ActF assay, fibrin network formation without platelet and thrombin contribution is assessed in the presence of reptilase/factor XIIIa (an activator of fibrin network formation) and abciximab (a GP IIb/IIIa inhibitor and anti-platelet agent) using heparinized blood. This assay measures MA_ActF_. The primary outcome of this study was the correlations between the early parameters and MA_HKH_ in the HKH assay of TEG6s Platelet Mapping.

Statistical analysis

Continuous variables are expressed as the median (interquartile range (IQR)), while categorical variables are expressed as raw numbers. Spearman’s rank correlation was used to assess the relationship between the parameters of TEG6s Platelet Mapping. The relationship between these parameters, fibrinogen concentration, and platelet count was further assessed using Spearman’s rank correlation. The strength of the relationship was assessed based on the correlation coefficient as negligible (correlation coefficient of 0.00-0.10), weak (0.10-0.39), moderate (0.40-0.69), strong (0.70-0.89), and very strong (0.90-1.00) [16]. To assess the ability of K, angle, and MAHKH to predict the combination of fibrinogen > 150 mg/dL [11,12] and platelet count > 100,000/µL [12,13], receiver operating characteristic (ROC) curves were drawn, and the area under the curve (AUC) was calculated. Optimal cut-off values were further calculated using Youden’s index [17]. Correlation coefficients and AUCs are expressed with 95% CI. Spearman’s rank correlation analysis was performed using GraphPad Prism 9 (GraphPad Software, La Jolla, CA, USA). ROC curves were created, and AUCs and cut-off values were calculated using EZR (Saitama Medical Centre, Jichi Medical University, Saitama, Japan). Statistical significance was set at P < 0.05.

## Results

Twenty-three patients underwent TEG6s Platelet Mapping at least once during surgery (Table 1), and 62 platelet mapping assays were performed (20 assays during CPB and 42 assays consisting of 20 assays at baseline and 22 assays at the end of surgery during the non-CPB period).

**Table 1:** Patient characteristics Data are presented as raw numbers or median (IQR). IQR, interquartile range; Ht, height; BW, body weight; CPB, cardiopulmonary bypass; CABG, coronary artery bypass grafting.

	N = 23
Age, years old	72 (66-79)
Gender (female/male), n	8/15
Ht, cm	160 (158-168)
BW, kg	56 (49-73)
BSA, kg/m^2^	1.60 (1.49-1.77)
Operation time, minute	429 (349-482)
CPB time, minute	224 (166-250)
Type of surgery, n	
Valve	3
CABG	1
Aortic	12
Complex	7

Overall, 59 pairs of data (17 pairs during CPB and 42 pairs consisting of 20 pairs at baseline and 22 pairs at the end of surgery during the non-CPB period) were available for the comparison between Platelet Mapping data and the data from the clinical laboratory (fibrinogen concentration and platelet count). Antiplatelet drugs were discontinued and replaced with heparin preoperatively in six patients and not discontinued due to emergency surgery in three patients.

**Figure 2:**
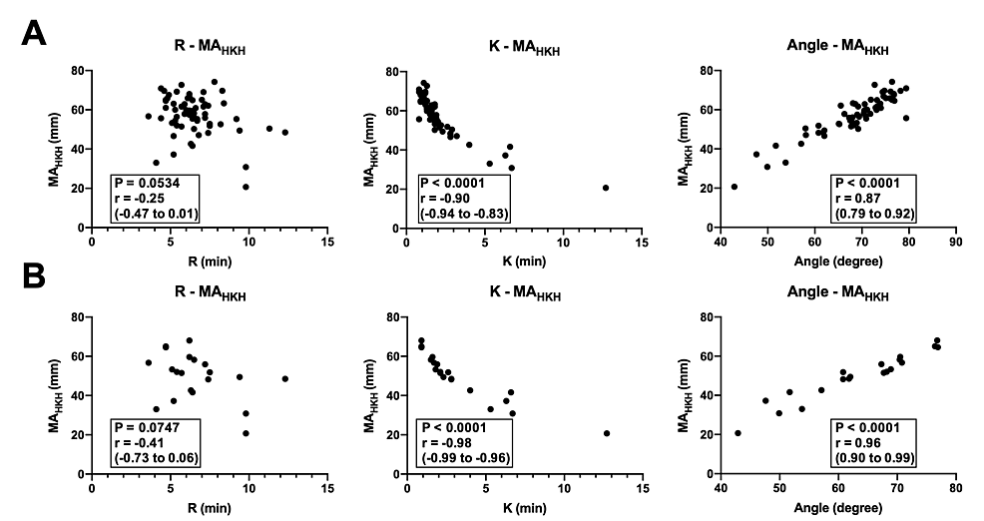
Correlation between MAHKH and early parameters in the HKH assay of TEG6s platelet mapping The figure shows Spearman’s rank correlation coefficient (r) expressed with a 95% CI. (a) All samples. (b) Heparinized blood samples obtained during CPB.

We first examined the relationship between MA_HKH_ and early parameters, including R, K, and angle, in the HKH assay of TEG6s Platelet Mapping. Although R was not significantly correlated with MA_HKH_, a very strong relationship between K and MA_HKH_ and a strong relationship between angle and MA_HKH_ were observed (r (95% CI): -0.90 (-0.94 to -0.83), p < 0.0001 for K, and 0.87 (0.79 to 0.92), p < 0.0001 for angle; Figure 2A). These relationships were further confirmed in the fully heparinized blood samples obtained during CPB (r (95% CI): -0.99 (-0.99 to -0.96), p < 0.0001 for K, and 0.96 (0.90 to 0.99), p < 0.0001 for angle; Figure 2B) with very strong relationships. These results indicate that K and angle, which can be obtained in the early phase of the HKH assay, are useful parameters for predicting the MA_HKH _value, which plays a central role in the assessment of thromboelastography.

**Figure 3:**
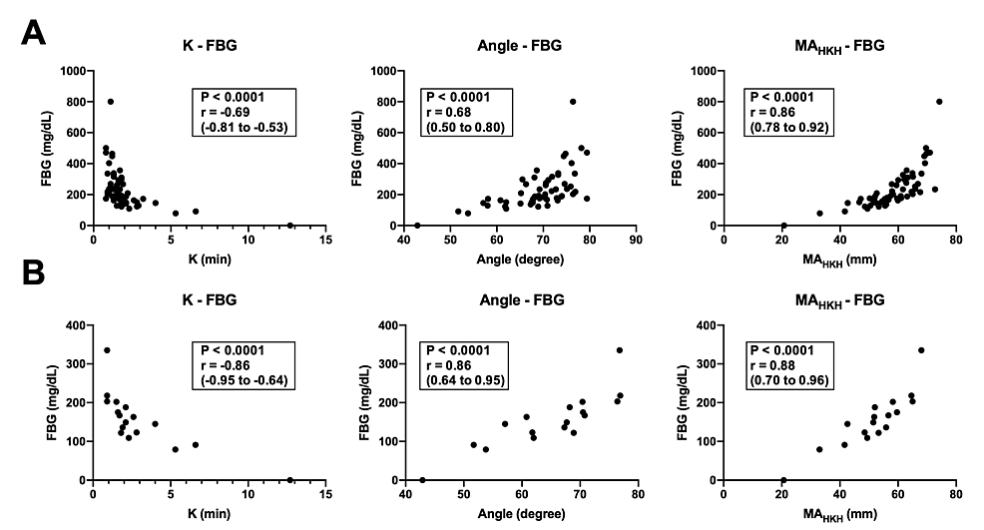
Correlation between fibrinogen concentration and each parameter in the HKH assay The figure shows Spearman’s rank correlation coefficient (r) expressed with a 95% CI. (a) All samples. (b) Heparinized blood samples obtained during CPB. FBG, fibrinogen.

Focusing on K, angle, and MA_HKH_, we assessed the relationship between these parameters and fibrinogen concentration and platelet count obtained from the clinical laboratory. Regarding fibrinogen, MA_HKH_ showed a strong relationship with fibrinogen concentration (r (95% CI): 0.86 (0.78 to 0.92), p < 0.0001; Figure 3A). Although the relationship was moderate, K and angle were still significantly correlated with fibrinogen concentration (r (95% CI): -0.69 (-0.81 to -0.53), p < 0.0001 for K, and 0.68 (0.50 to 0.80), p < 0.0001 for angle; Figure 3A). In the fully heparinized blood samples obtained during CPB, K, angle, and MA_HKH_ had strong relationships with fibrinogen concentration (r (95% CI): -0.86 (-0.95 to -0.64), p < 0.0001 for K, 0.86 (0.64 to 0.95), p < 0.0001 for angle, and 0.88 (0.70 to 0.96), p < 0.0001 for MA_HKH_; Figure 3B).

**Figure 4:**
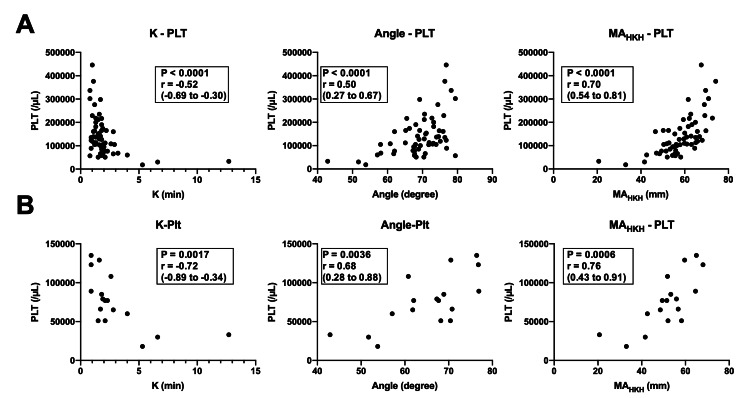
Correlation between platelet count and each parameter in the HKH assay The figure shows Spearman’s rank correlation coefficient (r) expressed with a 95% CI. (a) All samples. (b) Heparinized blood samples obtained during CPB. PLT, platelet.

Regarding platelets, as well as the results of fibrinogen, MA_HKH_ showed a strong relationship with platelet count (r (95% CI): 0.70 (0.54 to 0.81), p < 0.0001; Figure 4A). Although the relationship was moderate, K and angle were also significantly correlated with platelet count (r (95% CI): -0.52 (-0.69 to -0.30), p < 0.0001 for K, and 0.50 (0.27 to 0.67), p < 0.0001 for angle; Figure 4A). In the fully heparinized blood samples obtained during CPB, strong relationships of K and MA_HKH_ and a moderate relationship of angle with platelet count were observed (r (95% CI): -0.72 (-0.89 to -0.34), p = 0.0017 for K, 0.68 (0.28 to 0.88), p = 0.0036 for angle, and 0.76 (0.43 to 0.91), p = 0.0006 for MA_HKH_; Figure 4B).

**Figure 5:**
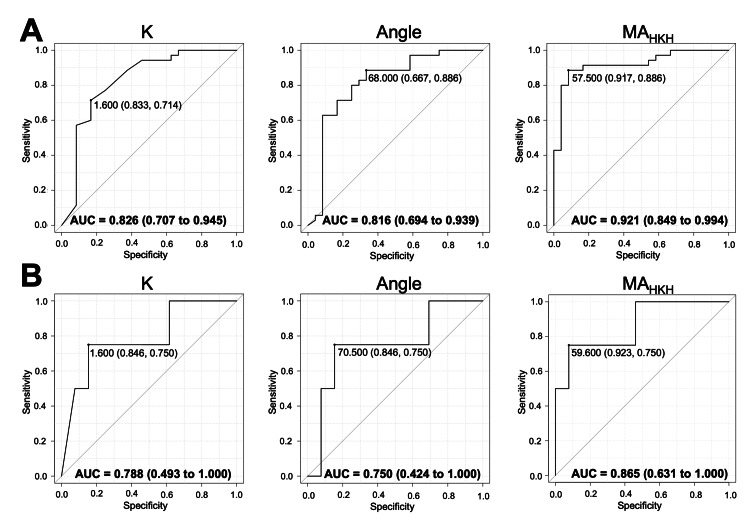
ROC curves of each parameter in HKH assay for combination of fibrinogen concentration > 150 mg/dL and platelet count > 100,000/µL AUCs are expressed with a 95% CI in the figure. (a) All samples. (b) Heparinized blood samples obtained during CPB. The cut-off value (specificity, sensitivity) is expressed in the figure.

Since the HKH assay reflects the overall contribution of both platelet and coagulation factors, including fibrinogen, we further drew ROC curves for each parameter to predict the combination of fibrinogen > 150 mg/dL and platelet count > 100,000/µL. According to the AUCs, K (AUC (95% CI): 0.826 (0.707-0.945)), angle (AUC (95% CI): 0.816 (0.694-0.939)), and MA_HKH_ (AUC (95% CI): 0.921 (0.849-0.994)) all showed a clinically acceptable ability to predict fibrinogen > 150 mg/dL and platelet count > 100,000/µL (Figure 5A). The cut-off values (specificity and sensitivity) of K, angle, and MA_HKH _are 1.6 min (83.3%, 71.4%), 68.0 degrees (66.7%, 88.6%), and 57.5 mm (91.7%, 88.6%), respectively. The positive predictive value and negative predictive value of these cut-off values are 86.2% and 66.7% for K, 79.5% and 80.0% for angle, and 93.9% and 84.6% for MA_HKH_, respectively. In the analysis with the heparinized blood samples during CPB (Figure 5B), although the AUCs of each parameter with the heparinized blood samples (AUC (95% CI): 0.788 (0.493-1.000) for K, 0.750 (0.424-1.000) for angle, and 0.865 (0.631-1.000) for MA_HKH_) are slightly smaller than those in the analysis of all samples, similar results were confirmed in this subgroup.

## Discussion

The present study revealed that K and angle, which are early parameters in the HKH assay of TEG6s Platelet Mapping, had a strong relationship with MA_HKH_, even in heparinized blood samples collected during CPB, suggesting the predictive value of K and angle for MA_HKH_. Additionally, all of K, angle, and MA_HKH_ had a significant ability to predict the combination of fibrinogen > 150 mg/dL [11,12] and platelet count > 100,000/µL [12,13].

The usefulness of TEG6s Platelet Mapping in cardiovascular surgery has been a topic of discussion recently because Platelet Mapping can be conducted using heparinized blood samples during CPB. A study examining the validity of the TEG6s assay during CPB using citrated blood samples activated with kaolin was previously reported. The authors revealed that despite the presence of HKH in this assay, the assay results were unreliable because of inadequate neutralization of heparin [18]. In contrast, another recent report examining the utility of TEG6s Platelet Mapping during CPB showed that MA_ActF_ could predict fibrinogen concentration with high accuracy during both the CPB and non-CPB periods. This study suggests that HKH in TEG6s Platelet Mapping fully neutralizes heparin in the blood sample during CPB [9]. Although data to clarify the maximum concentration of heparin that can be antagonized by the HKH in TEG6s Platelet Mapping is still lacking, the results of our study and the previous study [9] indicate that TEG6s Platelet Mapping can be used to evaluate patients’ coagulation status, even during CPB, in most clinical situations.

The ActF assay examines fibrin network formation without platelet and thrombin contributions. Our results concerning the predictive value of MA_ActF_ for fibrinogen concentration during CPB (data is not shown) also agree with those of a previous report [9]. However, the in vivo coagulation process is an elaborate orchestra that involves platelets and coagulation factors, including fibrinogen. Therefore, in addition to the independent assessment of fibrinogen concentration, it is critical to assess the overall coagulation status involving both platelets and coagulation factors in clinical practice.

HKH assay in TEG6s Platelet Mapping is initiated by kaolin and represents the overall contribution of thrombin-mediated fibrin formation and platelet aggregation. Therefore, this assay is considered a critical test in the assessment of patients’ global coagulation status. In thromboelastography, including the HKH assay, MA is the most critical parameter as it represents the maximal clot strength. Indeed, MA has been reported to be significantly correlated with postoperative blood loss after cardiac surgery [10], and MA plays a central role in thromboelastography-guided algorithms for blood coagulation management in cardiac surgery [19-22]. However, the time required to measure MA may limit the utility of this assay because patients’ coagulation status shows dynamic changes in a very short period, especially in cardiovascular surgery. This limitation allowed us to explore another early parameter that could predict MA.

R, K, and angle are all fast, early parameters in the HKH assay of TEG6s Platelet Mapping which represent the speed of the clot formation. Notably, according to the results of the present study, K and angle showed strong correlations with MA_HKH_ in both heparinized and non-heparinized blood samples. These results enable us to rapidly assess the overall coagulation status of the patients using K and angle. In contrast, R did not show a significant correlation with MA_HKH_, indicating that the initiation state of the coagulation process does not correlate with the maximum clot strength in the final phase of coagulation. This might be explained by the fact that according to the cell-based model of hemostasis [8], the initiation phase does not occur independently on the surface of platelets. However, the detailed mechanism remains unclear.

The present study also evaluated the relationships between Platelet Mapping parameters and conventional coagulation tests. MA_HKH _showed a strong relationship with fibrinogen concentration and platelet count, although K and angle showed moderate relationships with them. These results indicate that the predictive ability of TEG6s Platelet Mapping for conventional coagulation tests is comparable to that of rotational thromboelastometry (ROTEM; TEM Innovations Gmbh, Munich, Germany), which is another popular POC monitoring [23-25]. Furthermore, our study showed that K, angle, and MA_HKH_ could predict the combination of fibrinogen > 150 mg/dL and platelet count > 100,000/µL with clinically acceptable accuracy. As shown in the cell-based model of hemostasis [8], blood coagulation in vivo is a complex process involving the interaction of different types of cells and coagulation factors. Therefore, in addition to the independent assessment of each factor, the assessment of overall blood coagulation is critical for clinical practice.

Additionally, it is important that these HKH assay parameters can save valuable time compared to the examination of the fibrinogen and platelet count at the clinical laboratory. While it takes only around eight minutes to measure K, which is the earliest parameter in K, angle, and MA, it usually takes more than 30 minutes from sample collection to receive final results from the clinical laboratory, suggesting a significant advantage of TEG6s assessment in terms of time-saving during surgery. The authors believe that these results could provide clinicians with practical information to guide rapid decision-making regarding transfusion strategies during cardiac surgery.

The present study has several limitations. First, we did not examine the association between the results of Platelet Mapping and patients’ outcomes, such as the amount of bleeding and blood product, because of the relatively small number of samples and the wide range of patient characteristics. However, since the aim of this study was to evaluate the relationship between MA_HKH_ and the other early parameters in the HKH assay of TEG6s Platelet Mapping and the predictive ability of these parameters for the combination of certain levels of fibrinogen concentration and platelet count, the authors consider this to be beyond the scope of this research. A future prospective study is needed to validate the utility of the early parameters of the TEG6s Platelet Mapping-guided transfusion algorithm to improve clinical outcomes including patient prognosis and reduce the costs of transfusion in cardiovascular surgery. Second, because this was a retrospective study, we did not assess how the results of TEG6s Platelet Mapping affected the clinician’s decision-making in coagulation management during surgery. Finally, although TEG6s Platelet Mapping is generally conducted using whole blood, the actual coagulation process in vivo may be different because of the existence of other factors, including blood flow and endothelial cells.

## Conclusions

In summary, our results show that K and angle in the HKH assay of TEG6s Platelet Mapping are strongly correlated with MA_HKH_, even during CPB, and that these parameters all predict a combination of certain levels of serum fibrinogen concentration and platelet count with clinically acceptable accuracy. These findings suggest that not only MA_KHK _but also K and angle, which are early parameters in the HKH assay, provide clinically significant information that will facilitate rapid decision-making regarding coagulation strategies during cardiovascular surgery including the CPB period.
